# Kunitz-Type Peptide HCRG21 from the Sea Anemone *Heteractis crispa* Is a Full Antagonist of the TRPV1 Receptor

**DOI:** 10.3390/md14120229

**Published:** 2016-12-15

**Authors:** Margarita Monastyrnaya, Steve Peigneur, Elena Zelepuga, Oksana Sintsova, Irina Gladkikh, Elena Leychenko, Marina Isaeva, Jan Tytgat, Emma Kozlovskaya

**Affiliations:** 1 G.B. Elyakov Pacific Institute of Bioorganic Chemistry, Far Eastern Branch, Russian Academy of Sciences, 159, Pr. 100 let Vladivostoku, Vladivostok 690022, Russia; zel01@mail.ru (E.Z.); sintsova0@gmail.com (O.S.); irinagladkikh@gmail.com (I.G.); 969844@gmail.com (E.L.); issaeva@gmail.com (M.I.); kozempa@mail.ru (E.K.); 2 Toxicology and Pharmacology, University of Leuven (KU Leuven), Campus Gasthuisberg O&N2, Herestraat 49, P.O. Box 922, Leuven B-3000, Belgium; Steve.Peigneur@pharm.kuleuven.be (S.P.); jan.tytgat@pharm.kuleuven.be (J.T.)

**Keywords:** sea anemone, Cnidaria, TRPV1, Kunitz-type peptides, electrophysiology

## Abstract

Sea anemone venoms comprise multifarious peptides modulating biological targets such as ion channels or receptors. The sequence of a new Kunitz-type peptide, HCRG21, belonging to the *Heteractis crispa* RG (HCRG) peptide subfamily was deduced on the basis of the gene sequence obtained from the *Heteractis crispa* cDNA. HCRG21 shares high structural homology with Kunitz-type peptides APHC1–APHC3 from *H. crispa*, and clusters with the peptides from so named “analgesic cluster” of the HCGS peptide subfamily but forms a separate branch on the NJ-phylogenetic tree. Three unique point substitutions at the N-terminus of the molecule, Arg1, Gly2, and Ser5, distinguish HCRG21 from other peptides of this cluster. The trypsin inhibitory activity of recombinant HCRG21 (rHCRG21) was comparable with the activity of peptides from the same cluster. Inhibition constants for trypsin and α-chymotrypsin were 1.0 × 10^−7^ and 7.0 × 10^−7^ M, respectively. Electrophysiological experiments revealed that rHCRG21 inhibits 95% of the capsaicin-induced current through transient receptor potential family member vanilloid 1 (TRPV1) and has a half-maximal inhibitory concentration of 6.9 ± 0.4 μM. Moreover, rHCRG21 is the first full peptide TRPV1 inhibitor, although displaying lower affinity for its receptor in comparison with other known ligands. Macromolecular docking and full atom Molecular Dynamics (MD) simulations of the rHCRG21–TRPV1 complex allow hypothesizing the existence of two feasible, intra- and extracellular, molecular mechanisms of blocking. These data provide valuable insights in the structural and functional relationships and pharmacological potential of bifunctional Kunitz-type peptides.

## 1. Introduction

Sea anemone (phylum Cnidaria, class Anthozoa) venom is a rich source of proteinaceous toxins acting on ion channels such as voltage-gated sodium and potassium channels, and the TRPV1 [1,2,3,4,5] as well as a lot of protease inhibitors belonging, mainly, to a numerous BPTI/Kunitz-type family [6]. The peptides of this family have an ancient Kunitz fold and some of them are characterized by a unique and intriguing feature of dual functionality since they inhibit both proteases and ion channels [7,8,9,10]. This phenomenon is probably caused by the selection of peptide structures during divergent evolution together with specialization, diversification and sub- and neofunctionalization [11]. Considering an increasing diversity in sea anemone preys and predators, more than one function in the same peptide scaffold is advantageous for the survival of these organisms on Earth for nearly 500 million years [12]. It is believed that Kunitz peptides in sea anemone venom protect the own toxins from protease degradation and at the same time, paralyze a prey through inhibition of ion channels [9,10].

To date, a number of highly homologous native serine protease inhibitors has been isolated from different species: *Anemonia sulcata* [7,13], *Heteractis crispa* [14,15,16,17,18], *Anthopleura aff. xanthogrammica* [19,20], *Anthopleura fuscoviridis* [21], *Stichodactyla helianthus* [22,23,24], and *Stichodactyla haddoni* [8]. Three Kunitz-type protease inhibitors, first representatives of bifunctional molecules, AsKC1–AsKC3 [7], SHTX III [8], APEKTx1 and ShPI-1 [9,10], belonging to the structural type 2 sea anemone toxins, have been shown to exhibit both a powerful blocking activity against voltage-gated potassium channels and a serine protease inhibitory activity. Moreover, ShPI-1 has broad protease inhibitory specificity as it inhibits not only serine but also cysteine and aspartate proteases [22].

It was found that *H. crispa* Kunitz-type protease inhibitors are coded by the multigene superfamily and form a combinatorial library including HCGS, HCRG, HCGN, and HCGG peptide subfamilies [11]. The HCGS peptide subfamily and their evolutionary relationships were described in details previously [11,25]. Two sea anemone trypsin inhibitors, RmInI and RmInII, possess antihistamine activity in vivo [15]. Recently, we have shown that rHCGS1.20 has an anti-inflammatory activity thanks to its ability to reduce the content of nitric oxide (NO) in lipopolysaccharide activated macrophages. Moreover, this peptide, as well as rHCGS1.19 and rHCGS1.36, possess antihistamine activity by inhibiting the increase of the concentration of calcium ions in mouse bone marrow derived macrophages [26,27]. HCRG1 and HCRG2 are the first two representatives of a new Kunitz-type HCRG peptide subfamily [18]. These peptides are more potent inhibitors of trypsin and α-chymotrypsin than some known representatives of the HCGS subfamily [14,16,17] and they have exhibited an anti-inflammatory activity through inhibition of inflammatory mediators [18].

It was also found that three *H. crispa* serine protease inhibitors, APHC1, APHC2, and APHC3, possess an analgesic activity [28,29,30,31] by exhibiting an inhibitory activity against the pain receptor TRPV1 [28,32]. They are the first sea anemone peptide characterized as TRPV1 modulators. APCH1 decreases the capsaicin induced currents through TRPV1 with 32% ± 9% (EC_50_ = 54 ± 4 nM) [28]. Interestingly, the pharmacological potential of APHC1 and APHC3 can be considerably expanded by their hypothermic effect [30], which is not typical for low molecular weight TRPV1 antagonists.

Among all known ion channels involved in the regulation of a variety of intracellular signaling pathways, perception, and conduction of pain signals in dorsal root ganglia (DRG) neurons, an important integrator of painful and inflammatory stimuli is the TRPV1 receptor [33,34]. It belongs to the family of polymodal TRP channels, serving as a molecular cellular sensor, which is activated by a wide spectrum of physical and chemical stimuli [35,36,37]. They have diverse biophysical properties such as cation selectivity, specific mechanisms of activation, and they play the main role in many physiological processes—fertilization, development, cell survival, sensory transduction, etc. [38,39]. The TRPV1 receptor is a promising biological target for searching new analgesic agents as well as therapeutic target for various pain states [33,36,37,40]. It is believed that, unlike traditional analgesic agents (aspirin, paracetamol/acetaminophen, and other non-steroidal anti-inflammatory drugs) that suppress or treat inflammatory processes or the transmission of pain signals, TRPV1 antagonists prevent pain by inhibiting the receptor on susceptible neurons [41,42]. Currently, many low molecular weight TRPV1 agonists as well as antagonists have been studied [43] (some of them are already used or will be used in clinical practice [44,45]). It should be noted that the use of TRPV1 antagonists as analgesic agents until now is hindered by their significant side effects, mainly propensity to induce hypothermic effects [41,42,44]. Notably, abundant scientific research has focused on the development of approaches to overcome these side effects. Focusing on the nature of the TRPV1 receptor itself—multimodality with respect to different stimuli and the selection of successful combination of such factors of TRPV1 regulation as antagonist, effective dose, pH, temperature control, way of delivery, etc. [46,47]—will certainly contribute to the progress in developing antagonists suitable for clinical practice. There are only five venom-derived peptides acting on TRPV1 known up to date. A double-knot toxin DkTx, from the Chinese bird spider *Ornithoctonus huwena* [48], the toxins VaTx1–VaTx3 from the tarantula *Psalmopoeus cambridgei* [49], and BmP01 from the scorpion *Mesobuthus martensii* [50] are agonists, while APHC1–APHC3, from the sea anemone *H. crispa*, are three partially antagonists of the TRPV1 receptor. [28,30,31]. Sea anemone Kunitz-type peptides can be considered as a valuable instrument for studying the structure and function of TRP channels and as a template for designing novel analgesic agents [30].

Here, we describe a new Kunitz-type bifunctional peptide, representative of the *H. crispa* HCRG peptide subfamily, which is the first full antagonist of TRPV1 receptor.

## 2. Results and Discussion

### 2.1. cDNA hcrg21 Gene and Recombinant Peptide Obtaining

**Figure 1:**
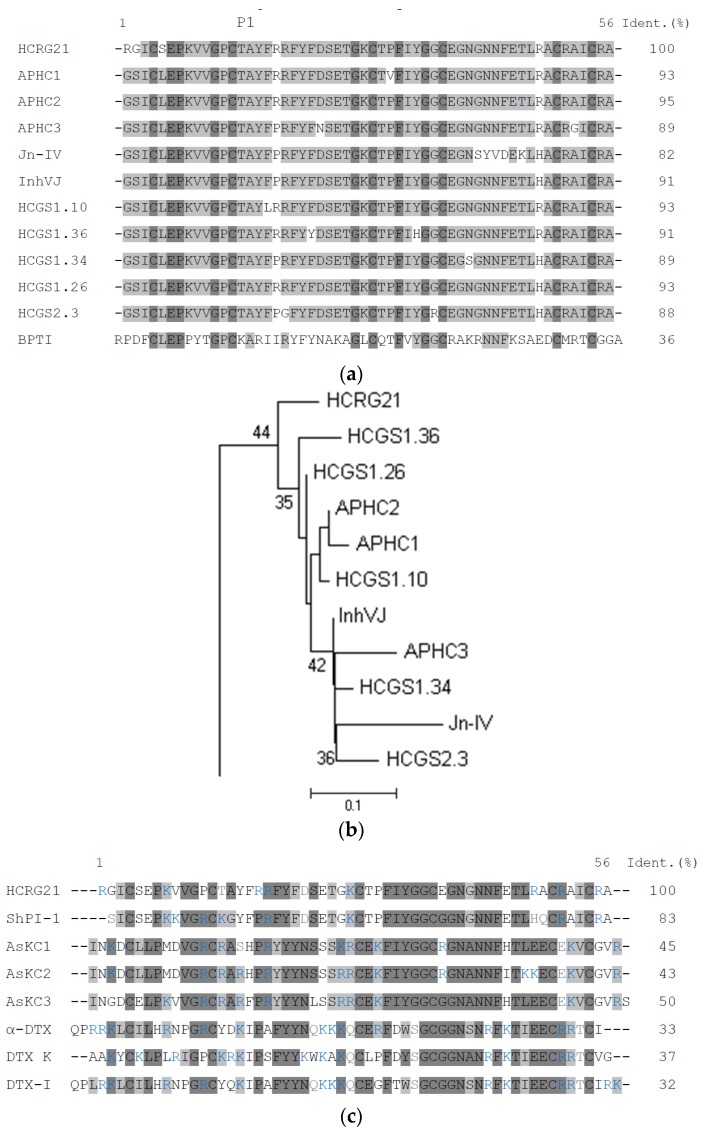
(**a**) Alignment of *H. crispa* Kunitz-type peptides amino acid sequences. Analgesic peptides, APHC1, APHC2, APHC3 [28,29], Jn-IV [14], InhVJ [16], and representatives of HCGS peptide family with P1Thr from the combinatorial library, obtained from *H. crispa* cDNA [11]: HCGS1.10, HCGS1.36, HCGS1.34, HCGS1.26, and HCGS2.3; and BPTI from bovine pancreas [55]. P1—amino acid residue of the inhibitor reactive center. Identical and conservative residues are indicated by dark and light gray colors. The asterisks below the sequence of BPTI indicate the contact sites with serine proteases; (**b**) Evolutionary relationships of *H. crispa* sea anemone Kunitz-type peptides. The NJ phylogenetic tree was made using the Poisson correction with bootstrap test of 1000 replications in MEGA 6 [56]. Nodes with confidence values greater than 30% are indicated; (**c**) Comparison of amino acid sequences of *H. crispa* HCRG21, ShPI-1 from *S. helianthus* [22], AsKC1–AsKC3 from *A. sulcata* [7] and α-DTX, DTX-K, and DTX-I from the snake *Dendroaspis polylepis* [53]. Identical and conservative residues are indicated by dark and light gray colors, respectively. Positive charged residues are colored in blue.

To study structural diversity of a new HCRG peptide subfamily, a combinatorial library of HCRG peptides was obtained [51]. Here for, nested PCR with gene specific primers created on the basis of nucleotide sequences of Kunitz-type peptide genes was used [11] (Table S1). Analysis of the deduced amino acid sequences revealed that all peptides have a N-terminal Arg1 and Lys14 at the P1 position. However, one unique peptide, HCRG21, has Thr14 at this position, similar to the representatives of so named “analgesic cluster” of HCGS peptide subfamily have. This cluster is composed of analgesic peptides APHC1–APHC3 which are also known as modulators of the TRPV1 receptor [28,30,31,32]. HCRG21 shares 82% to 95% of identity with APHC1–APHC3, as well as with Jn-IV, InhVJ, and some HCGS peptides deduced by the PCR-based cloning technique (HCGS1.10, HCGS1.34, HCGS1.36, HCGS1.26, and HCGS2.3) (Figure 1a) [11]. All these peptides slightly differ from each other. Therefore, it is not surprising that, according to phylogenetic analysis, HCRG21 clusters with the analgesic peptides but forms a separate branch on the neighbor-joining (NJ) phylogenetic tree (Figure 1b). It should be noted that three unique point substitutions at the N-terminus of the molecule, Arg1, Gly2, and Ser5, differ between HCRG21 and other peptides of this cluster.

Moreover, HCRG21 is highly homologous, 84% of identity, with ShPI-1, a protease inhibitor from the sea anemone *S. helianthus*, which binds with high affinity to Kv1.1, Kv1.2, and Kv1.6 channels [10]. For many potent Kv channel blocking toxins, such as α-DTX, DTX-K, and DTX-I, isolated from the snake *Dendroaspis polylepis,* it has been well established that mainly a series of positive charged residues (for example, R3, R4, K5, K37, K39, K46, and K50 in α-DTX) are crucial for interaction with the Kv channel [52,53,54]. In addition, for Kv channel toxins from sea anemones, such as the kalicludines from *A. sulcata*, AsKC1 and AsKC2, a N-terminal dyad (K3/L7 in AsKC1 and AsKC2) together with a number of non-polar residues have been identified as important for peptide binding with the channels [7]. Amino acid sequence alignment of Kv blockers from the snake, α-DTX, DTX-K, and DTX-I, and the sea anemones, ShPI-1 and AsKC1, and HCRG21 (Figure 1c) demonstrates the presence of positive charged residues (8 in HCRG21 and ShPI-1, 10 in AsKC1, in comparison with 11 in DTX-K and 14 α-DTX, and DTX-I) located not only at the N- and C-terminus, but throughout the whole sequence.

Based on its sequence, HCRG21, could exhibit protease, Kv and/or TRPV1 inhibitory blocking activity. To verify this assumption, the recombinant peptide, rHCRG21, was obtained and functionally tested.

**Figure 2:**
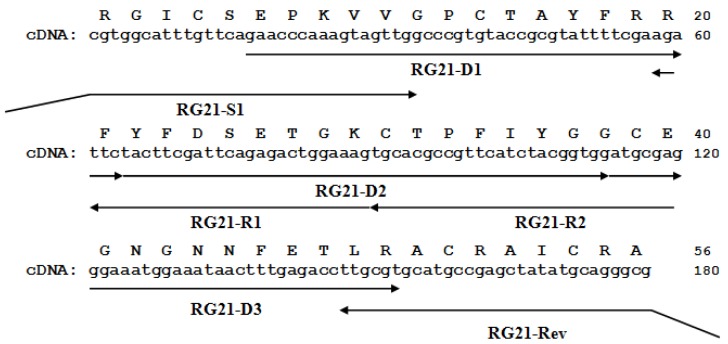
The *hcrg21* gene sequence and the deduced amino acid sequence of HCRG21. The arrows show the direction and length of the primers used for gene synthesis. The digits on the right show the length of the nucleotide and amino acid sequences.

**Figure 3:**
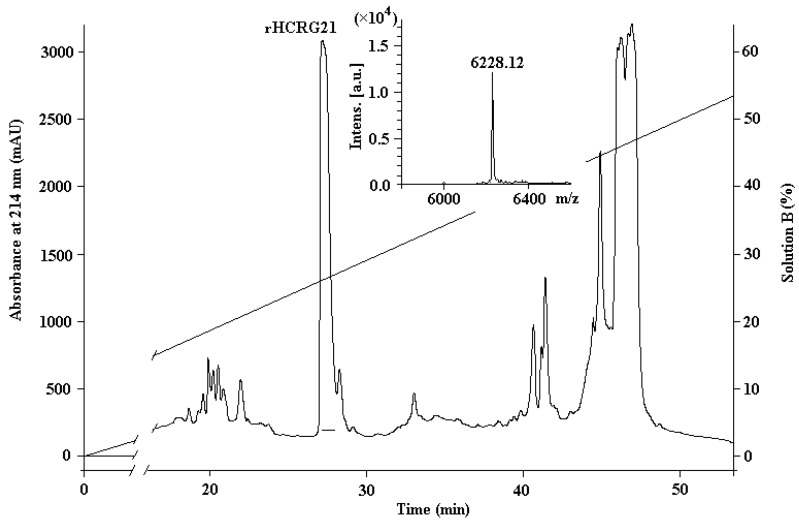
The RP-HPLC elution profile of rHCRG21, obtained as the result of hydrolysis of the fusion protein Trx-HCRG21 by BrCN, on a Jupiter C4 column (Phenomenex, Torrance, CA, USA), equilibrated by 0.1% TFA, pH 2.2, (Solution A), in a gradient of acetonitrile (Solution B) concentration (0%–70%) for 70 min at 3 mL/min. Fractions containing the mature peptide rHCRG21 are underlined. Insert: MALDI-TOF/MS spectrum and molecular mass of rHCRG21 after RP-HPLC.

Figure 2 shows the *hcrg21* cDNA including an open reading frame of 234 bp, which encodes 56 amino acids of the mature peptide, and a *hcrg21* gene synthesis scheme. The synthesis of the *hcrg21* gene was carried out by two PCR rounds using gene specific primers created on the basis of the nucleotide sequence with optimized codons according to *E. coli* rare codons (Table S1). rHCRG21 was produced as a fusion protein with a thioredoxin (Trx) domain with a molecular mass of about 25 kDa (according to electrophoresis data) and isolated from culture medium by metal affinity chromatography, cleaved by cyanogen bromide (CNBr), and purified by HPLC. The yield of the recombinant peptide was 5.5 mg/L of the cellular culture. Trypsin inhibitory activity was detected in the fraction marked rHCRG21 (Figure 3). According to MALDI-TOF/MS analysis, the molecular mass of rHCRG21 yielded 6228.12 Da (Figure 3 insert) which corroborated well the calculated theoretical mass when considering the formation of three disulfide bonds (6228.10 Da).

### 2.2. Comparison of Kunitz-Type Inhibitors Protease Inhibiting Activity

Similar to the most potent trypsin inhibitor, BPTI, many sea anemone peptides are very active towards trypsin because of the presence of functionally important P1 Lys or Arg residues at their reactive sites located at the canonical binding loop [57]. It is shown, that the first known *H. crispa* analgesic Kunitz-type peptides, APHC1–APHC3, have a Thr residue (instead of Lys or Arg) at position P1, similar to other representatives of the analgesic cluster (Figure 1a,b). Inhibition constants (*K_i_*) of rHCRG21 for trypsin and α-chymotrypsin determined by the Dixon method yielded 2.0 × 10^−7^ and 7.0 × 10^−7^ M, respectively (Table 1). It is known that interactions between trypsin-like proteases and serine protease inhibitors having branched residues, such as Thr, Val, or Ile, located at the P1 position is not favorable [58,59]. Therefore, in contrast with the affinity of BPTI to trypsin, many sea anemone Kunitz-type inhibitors such as InhVJ, Jn-IV, and APHC1–APHC3 display a (Figure 1b) significantly lower potency (in the range of *K*_i_ values 10^−6^–10^−9^ M). As such, the trypsin inhibitory activity is in line with other peptides of this subfamily. The constant values of trypsin inhibition for rHCRG21, APHC2 (9.0 × 10^−7^ M), and APHC3 (5.0 × 10^−7^ M) [29] are of the same magnitude orders. Nevertheless, a slightly higher value of *K_i_* for rHCRG21 compared to those of APHC1–APHC3, can be explained by the presence of a N-terminal Arg, which noticeably increases the energy of Kunitz-type *H. crispa* peptides binding with proteases. This energy benefit has been shown previously by computational methods for HCRG1 and HCRG2 [18] (Table 1).

At the same time, according to the previously proposed hypothesis [11], replacement of residues at position P1 (Arg → Lys → Thr) during evolution has resulted, along with the specialization of some Kunitz-type peptides, into diversification and sub- and neofunctionalization of ancestral peptides of this superfamily. Furthermore, it allowed the appearance of peptides with dual functionality, e.g., Kv and TRPV1 channel inhibitory activity together with trypsin inhibitory activity.

### 2.3. Activity to Kv and TRPV1 Channels: Electrophysiological Assay and Comparative Structure Analysis

In order to characterize HCRG21 functionally, recombinant HCRG21 was, at a concentration of 10 μM, tested for its activity against an 8 voltage-gated potassium channels (Kv1.1–Kv1.6, hERG, and *Shaker* IR) and the vanilloid receptor 1 (TRPV1).

**Figure 4:**
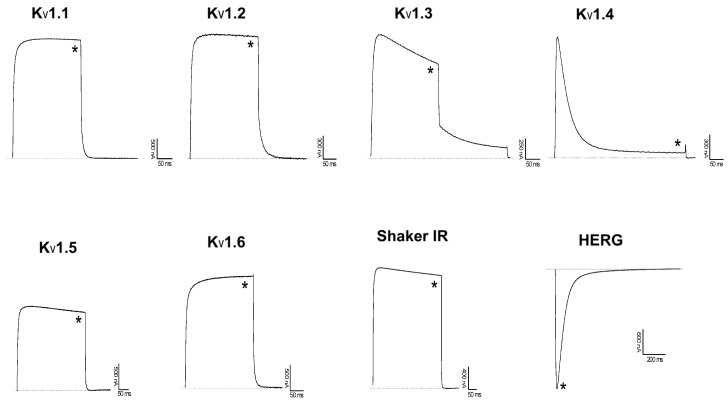
Activity of rHCRG21 on Kv channels expressed in *X. laevis* oocytes. Traces shown are representative of at least three independent experiments (*n* ≥ 3). The dotted line indicates the zero current level. The asterisk (*) distinguishes the steady-state current after application of 10 μM peptide.

However, rHCRG21 did not exert any activity on the Kv channels tested (Figure 4). Although there are many positively charged residues present in the sequence of HCRG21, they do differ in position with known Kv1.1–Kv1.6 blockers, such as α-DTX, DTX-K, DTX-I, AsKC1, and ShPI-1 (Figure 1c). Previously, an absence of Kv channel activity was also observed for the InhVJ [17]. Noteworthy, several positively charged residues are conserved between HCRG21 and ShPI-1 (in positions 8, 19, 28, 51, and 55, Figure 1c). Therefore, other Kv channel isoforms should be tested for their sensitivity to HCRG21 as well.

**Figure 5:**
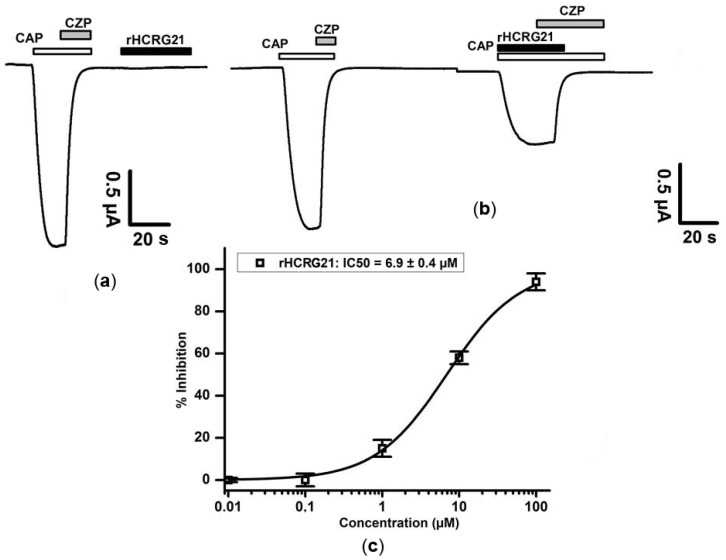
Activity profile of rHCRG21 (10 μM) on the TRPV1 receptor. The figures show the results of one representative experiment. Whole-cell current traces in control and rHCRG21 conditions are shown. (**a**) Lack of effect of rHCRG21 (10 μM) in the absence of capsaicin; (**b**) Antagonistic effect of HCRG21 co-applied with capsaicin (CAP = 2 μM); capsazepine (CZP = 10 μM). All data represent the average of at least three independent experiments (*n* ≥ 3) and are presented as mean ± standard error; (**c**) Concentration–response curve for rHCRG21 on TRPV1 receptor.

To control the expression level of the TRPV1 channels, experiments were initiated with a first activation of TRPV1 with 1 μM capsaicin (CAP). Application of capsaicin opens the channels. This activation gives an inward non-selective cation current. Subsequent application of capsazepine (CZP) closes the channels allowing the current levels to return to zero. Before application of HCRG21, cells are washed with ND96 to remove any presence of capsazepine. As for the interaction of rHCRG21 with TRPV1, when peptide was administered alone, it did not show any significant effect (Figure 5a). Similarly, no activity was seen when HCRG21 was applied prior to any stimulation of capsaicin (data not shown). However, when rHCRG21 was co-applied together with the agonist capsaicin, a reduction of the current was observed (Figure 5b). Application of 10 μM rHCRG21 caused an inhibition with 59.3% ± 4.3% (*n* ≥ 3). A concentration–response curve was constructed in order to assess the concentration dependence of the rHCRG21 induced effect. The IC_50_ value yielded 6.9 ± 0.4 μM (Figure 5c). Although rHCRG21 has apparently lower affinity than APHC1, which inhibits TRPV1 only partially (the highest percentage of inhibition obtained was ~50% [28]), there was almost full inhibition of the current through TRPV1 with a maximum observed inhibition of 95% (Figure 5c).

The difference in efficacy between rHCRG21 and APHC1 on TRPV1 (95% and 50% receptor inhibition, respectively) seems to result from small differences in functionally important basic residues within the sequence of both peptides. Previously, on the basis of experimental data [28] and computer modeling [32], it was suggested that the most functionally important residues are Glu6, Thr14, Val31, Glu38, Arg48, and Arg51 in APHC1. All these residues are conserved between APHC1 and HCRG21, except Val31, which is replaced by a Pro in HCRG21. It is well known that prolines can induce a structural kink in a peptide structure. It is possible that the Val31 to Pro substitution in HCRG21 results in a conformational change that allows HCRG21 to expose a contact surface differing from that of APHC1, which might explain the differences in efficacy between these peptides. However, detailed structural studies are required to verify this hypothesis.

### 2.4. Molecular Modeling

In this paper, we continued the study of the ligand-receptor interaction between HCRG21 and TRPV1 in order to clarify the mechanism of HCRG21 action. Application of modern bioinformatic approaches and programs of molecular modeling (molecular docking and MD simulations) allows to set clear patterns and to speculate about the subtle intermolecular interactions of ion channel or receptor and their antagonists/agonists, which represents the basis for the creation of novel selective pharmacophores for these targets.

Earlier, we have made an attempt to understand the mechanism, underlying the action of APHC1 from *H. crispa* on capsaicin-induced currents through the TRPV1 channel. Based on the homology model, we have made a conjecture that APHC1 may interact with the regulatory domain of intracellular receptor portion [60]. According to this model, a modulating effect of the peptide can be explained by its interaction with multiple (two or more) subunits of the regulatory receptor domain, which prevents its free transition from one state to another, and apparently extends the receptor relaxation time. Analysis of the architecture of the HCRG21 complex with the intracellular part of the receptor, derived from APHC1–TRPV1 complex [32] by homology docking and MD simulations have revealed that 26 HCRG21 residues, including Thr14, Tyr16 and Phe17, differing peptides of “analgesic cluster” (Figure 1b) from other *H. crispa* Kunitz-type peptides, are involved into hydrogen bonding and/or electrostatic interactions as well as π-interactions with all receptor subunits (in total 57 interactions, Table S2).

**Figure 6:**
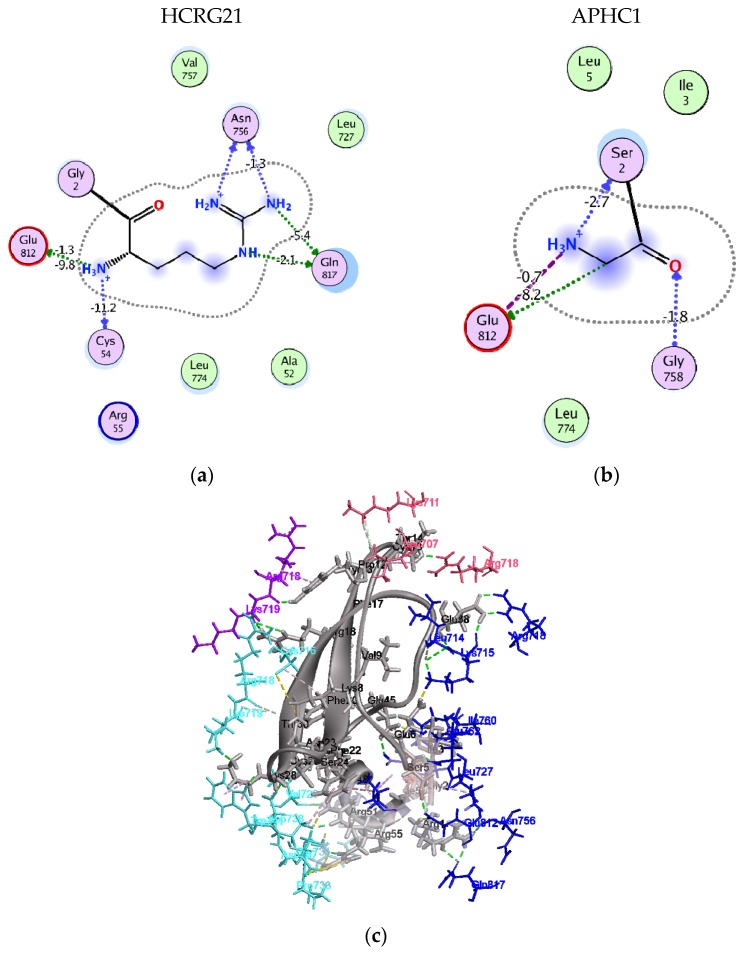
Molecular model of HCRG21 interaction with TRPV1 intracellular portion. 2D diagram of HCRG21 N-terminal residue interaction to TRPV1: (**a**) Arg1 of HCRG21; (**b**) Gly1 of APHC1; and (**c**) ribbon diagram of HCRG21 intermolecular interactions with TRPV1 regulatory domain. HCRG21 is represented as a ribbon, residues of both peptides and receptor involved in interactions are represented as sticks and colored according to subunits: direct hydrogen bonds colored in green; electrostatic and π-cation interactions in light brown; and hydrophobic interactions in magenta.

In agreement with this hypothesis, Arg1 contributes to binding energy of −20.1 kcal/mol compared to −10.7 kcal/mol for Gly1 of APHC1 (Figure 6a,b). In addition to backbone H-bonding and salt bridging to Glu812, its long basic and flexible side chain forms 4 hydrogen bonds to Gln817 and Asn756 of receptor subunit B. Contribution of HCRG21 Ser5 H-bonds to binding energy with Glu812 and Glu762 of the same receptor subunit was estimated as −2.8 kcal/mol. This observation clearly evidenced that replacement of Gly1 to Arg and Ley5 to Ser in HCRG21 further to Arg51 and Arg55 of APHC1 (and other analgesic peptides) enables HCRG21 to bind more tightly (compared to APHC1) the adjacent subunits of the receptor regulatory domain by two polar fingers (Figure 6c). This interaction manner is very similar to the one of DkTx, this peptide binds with the extracellular part of the receptor by hydrophobic aromatic fingers [61]. However, unlike the agonist DkTx, which causes a conformational change in the receptor upper pore region and enhances the ion current through the channel, HCRG21, most likely, hampers the transition of the receptor into another state and hereby the availability of the receptor for a following pain signal. This model obviously requires additional studies to investigate the ability of *H. crispa* Kunitz-type peptides to penetrate into cells.

This model is in good agreement with the analgesic action of “analgesic cluster” peptides in vivo that was demonstrated to be developing during 1–3 h [25,28]. Furthermore, a model of slow interaction also agrees with the data regarding kinetics of vanilloids penetration into the cell (equilibrium is reached near to 2 h) and herewith a dramatic decreasing of effective ligand concentration (by two orders of magnitude at 2 h of pre-incubation conditions) when compared to simultaneous application [62].

In the past four years, great progress has been made in the capabilities of cryoelectron microscopy. This provided new knowledge on the spatial organization of the extracellular and transmembrane domains of TRPV1 in absence of ligands, as well as in the low molecular agonist- and peptide agonist-bound states [61]. However, the available information about the rat TRPV1 minimal active fragment is still not enough for comprehension of how peptide antagonists or blockers may affect the receptor kinetics. For a deeper understanding of a possible mode of action of peptides on the pain vanilloid receptor, we performed a molecular modeling by exploring techniques of macromolecular docking and MD simulations.

**Figure 7:**
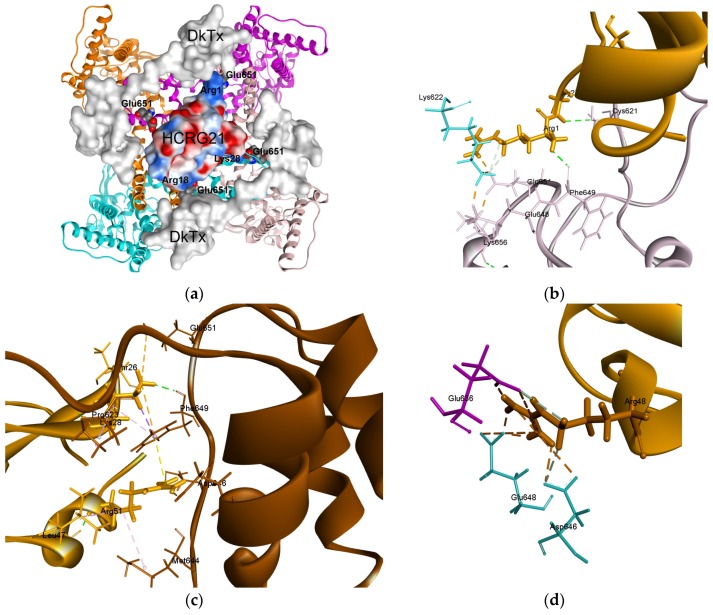

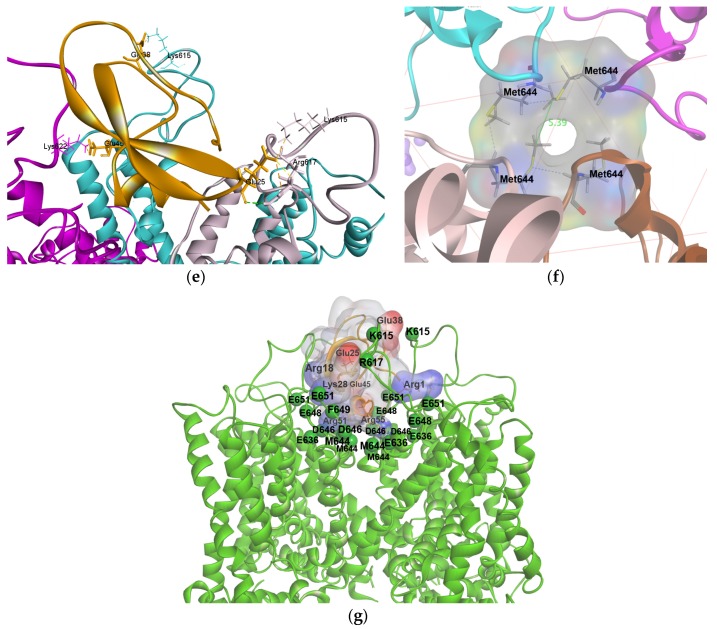
Structure model of complex of HCRG21 with TRPV1 receptor. (**a**) Superposition of HCRG21–TRPV1 complex to crioelectron microscope 3D-structure complex of DkTx with the receptor 5IRX [61]. Glu651 represented as bolds and colored according to elements, DkTx molecular surface is colored in gray, point distribution of molecular electrostatic potential (MEP) for HCRG21 molecule are presented; (**b**–**e**) Diagram of intermolecular non-bonded interactions in HCRG21–TRPV1 complex, several critical residues described in the text are represented as sticks and colored according to receptor subunits, HCRG21 residues colored in light brown. Intermolecular interactions as well as intramolecular ones are represented as dashed line and colored: hydrogen bonds in green; electrostatic and π-cation interactions in light brown; and hydrophobic in light magenta; (**f**) TRPV1 outer pore formed by Met644 residues in the TRPV1 complex with HCRG21 revealed by MD simulations. TRPV1 subunits are represented as a ribbon diagram and colored according to chain; side chains of Met644 are represented as sticks and colored according to elements; hydrogen bonds between adjacent receptor subunits are represented as blue dashed line; distance between Met644 side chains of opposite TRPV1 subunits is indicated; (**g**) The role of HCRG21 basic and acidic residues in interaction with TRPV1 pore and turret regions. HCRG21 is presented as ribbon diagram (light brown color) and molecular surface (light gray color), charged residues surfaces are colored according to extrapolated charge, positive in blue, negative in red. TRPV1 presents as ribbon diagram and colored in green. Localization of key TRPV1 turret and lining the pore residues involved into HCRG21 interaction is presented as bolls and marked by one letter amino acids code.

According to generated models, HCRG21 may bind in the area of the outer vestibule of the TRPV1 channel pore (Figure 7a). The peptide binding site does not overlap with known binding sites of vanilloids or small molecule agonists and antagonists. However, it is localized in close proximity to the binding site of the spider peptide toxin, DkTx, which binds with TRPV1 outer pore domain [48]. According to our docking data, Arg1, Arg18, and Lys28 of HCRG21 overlap with DkTx binding site only at the area of Glu651 residues of the TRPV1 receptor (Figure 7a).

The N-terminal polar residue Arg1 of HCRG21 binds tightly to both Phe649 and Glu651 of one subunit through multiple H-bonding and salt bridges with an estimated contribution to the binding energy of −12.27 kcal/mol and −16.82 kcal/mol, respectively. After MD simulations nearby Lys622 became bound to both Glu651 and Glu648 of adjacent subunit through hydrogen bond and strong ionic interactions. One can assume Arg1 to provide conformation of pore loop that allows acidic side chains of Glu651 and Glu648 to interact firmly (−43.10 kcal/mol) with the extracellular loop 3 through Lys622. At the same time the Gly2 backbone forms hydrogen bond with Cys621 of the same receptor subunit (Figure 7b). Probably this structural rearrangement highlights the role of the 1RG2 fragment, preventing the capsaicin provoked conformational changes at the pore loop region. From the other side, in a similar fashion, a basic Lys28 side chains of HCRG21 can electrostatically interact with Asp601 and Glu651 ones in addition to hydrogen bond with the Phe649 carbonyl oxygen of the opposite receptor subunit (Figure 7c) resulting in a total contribution to the binding energy of −10.19 kcal/mol. Beside this Arg51 brings the outer mouth of the channel pore through strong electrostatic interaction and hydrogen bonding with Glu636 (−18.29 kcal/mol) of the same subunit. Moreover, both Lys28 and Arg51 anchored strongly enough (with a total contribution of −9.70 kcal/mol) in the mouth of the pore by means of formation of π-interactions cluster across the Phe649 aromatic ring (Figure 7c).

According to this model, Arg48 of HCRG21 binds firmly two other TRPV1 subunits in the region of mouth of the pore by two H-bonds and multiple attractive electrostatic interactions with each of three acidic residues Glu648 (−22.76 kcal/mol) and Asp646 (−21.13 kcal/mol) of one receptor subunit as well as with Glu636 (−20.47 kcal/mol) of adjacent subunit (Figure 7d). At the same time, the Arg48 backbone is localized near the TRPV1 pore and can form a hydrogen bond with Met644 residue of third receptor subunit creating difficulties for an intracellular ion influx. It should be noted that the Glu648 residues are responsible for TRPV1 proton binding [63]. Interestingly, we have demonstrated previously that in contrast to APHC1 and HCRG21, peptide InhVJ, with Arg48 substituted by His, is inactive on the TRPV1 channel when co-applied with capsaicin [17]. In agreement with computational data, binding of InhVJ would bring a His residue in close proximity with receptor proton binding site (Glu648). Therefore, InhVJ could be a potential modulator of TRPV1 depending on the protonation state of His48 which is strongly depending on the environment.

MD simulations of the HCRG21–TRPV1 complex in a hydrated 1-palmitoyl-2-oleoyl-sn-glycero-3-phosphocholine (POPC) lipid bilayer showed that HCRG21 interaction with the extracellular portion of the receptor may cause a change in conformation in the area of the pore at the upper gate. Furthermore, it suggests a displacement of the pore loop (helix 630–642) and γ-turn 643–651 with RMSD ~1.68 Å for 22 Cα-atoms. Unlike the experimental frozen receptor structure, these rearrangements in the structure of the selectivity filter during MD simulations are not symmetrical among subunits. At the same time, this observation is consistent with reported results of a long microsecond time scale (1 μs) MD simulations of temperature activated TRPV1 [64]. MD simulations revealed that, upon complex formation with HCRG21, conformational changes in the pore loop region of the channel occur. These structural changes bring the side chains of Met644, which form the outer pore, in a sufficient close proximity (Figure 7e) to form hydrogen bonds (3.93 Å), that is not observed in a non-conducting receptor state (PDB ID: 3J5P). In the activated state of the channel, no intra- or inter-H-bonding in the pore helices are normally present [65,66]. Moreover, Ala52 of HCRG21 as well as methylene groups (-CH2-) of Arg48, Arg51, and Arg55 cooperate with Met644 residues from three channel pore loops to form a hydrophobic seal. Their contributions to complex stabilization were estimated at −1.91 kcal/mol, −1.57 kcal/mol, −1.45 kcal/mol, and −0.45 kcal/mol, respectively. As a result the solvent availability of residues forming the pore upper gate decreases from 25% in activated state to 10% in complex with HCRG21. This low solvent availability of the receptor pore agrees well with electrophysiological data on rHCRG21 action (Figure 7f).

MD simulations data showed also the role of acidic HCRG21 residues Glu25, Glu38, and Glu45 that seems like to interact with the TRPV1 pore turret (Figure 7f). These flexible extracellular loops were not determined experimentally but a pool of biochemical data evidenced its significance for TRPV1 responses on diverse stimuli [67,68]. Notably, these long extracellular loops that form TRPV1 pore turret and are directly linked to the channel pore helix represent a highly variable region among TRPV family members. The basic residues occurrence at the top of the turret (Lys615, Arg617, and Lys622) is characteristic for TRPV1 cannel isoform. These residues are omitted or replaced to neutral or acidic ones in other TRPV channels isoforms, except in human TRPV3 with only Lys622 in this loop [65].

Therefore, our modeling reconstruction confirms the crucial importance of positively charged residues ring (Arg1, Arg18, Lys 28, Arg48, Arg51, and Arg55) for HCRG21 action on current flow through ion channels that is discussed widely for those of Kv1 toxins [9,10,69]. Moreover, we ascertain the putative role of negatively charged residues in peptide antagonist interaction with TRPV1 channel. The former interact with acidic residues lining the TRPV1 pore and might penetrate deeply to the outer pore down to the close proximity for Met644 selective filter. The later possibly stabilize the complex by additional interaction of peptide antagonist with the TRPV1 extracellular loops 3, carrying a positive charge in the turret region (Figure 7g). The proposed model agrees with experimental data that rHCRG21 could fully inhibit the TRPV1 receptor with a maximum observed inhibition of 95%. In our computational simulations, we observed multiple interactions of peptide residues except Thr14 or Tyr16 with the residues of TRPV1 subunits at the outer vestibule. So, it remains unclear why the Thr residue at P1 position is of crucial importance for peptides of *H. crispa* “analgesic cluster” (Figure 1b). It should be also noted that peptides of this cluster unlike other members of HCGS and HCRG subfamilies have a very small net positive charge of the molecule (in range from 0 to +2) [32]. Moreover, they are characterized by a point distribution of the MEP [11,32]. It can be assumed that the specificity of a peptide modulator for its target may be well determined by the electrostatic complementarity between both. In this respect, the combinatorial library of *H. crispa*, which provided several Kunitz-type peptides with variations of single charged residues in their sequence, serves as a promising source of peptides specific to different receptor subtypes and various types of stimuli. Therefore, HCRG21 represents a promising tool for studying TRPV1 channels and, moreover, might be an interesting lead compound for the development of novel analgesics.

## 3. Materials and Methods

### 3.1. Gene Sequence Determination

cDNA library of Kunitz-type HCRG peptides was obtained by nested PCR (two round) with gene specific primers, created on the base of obtained nucleotide sequences of Kunitz-type peptide genes previously [11]. *H. crispa* cDNA obtained as reported [70] was used as matrix. The primer sequences and PCR conditions are shown in Table S1. The standard methods were used for cloning obtained PCR-fragments (~180 bp) and plasmid isolation [71]. Analyses of the nucleotide sequences of the inserts were performed on an ABI3130xl genetic analyzer (Applied Biosystems, Foster City, CA, USA) [72].

### 3.2. Synthesis of Gene Encoding HCRG21

Synthesis of the *hcrg21* gene was carried out by PCR (2 steps) using gene specific primers created on the base optimized according to rare codons for *E. coli* expression *hcrg 21* gene (Table S1). Expression construction was created by cloning the gel-purified PCR-fragment (~180 bp) into vector pET-32b(+) (Novagen) at the EcoRI and XhoI restriction sites by standard methods [71]. The resulted construct was checked by sequencing. The nucleotide and deduced amino acid sequences were analyzed using the Vector NTI 8 software package (Invitrogen, Grand Island, NY, USA).

### 3.3. Production and Physical and Chemical Characterization of Recombinant HCRG21

The transformed cells were cultured in LB medium (1 L) containing ampicillin (100 μg/mL) at 37 °C to the optical density of A_600_ 0.6–0.8. Isopropyl-β,d-thiogalactopyranoside (IPTG) was added to the final concentration of 0.2 mM for the expression induction. The cells were grown for 16–18 h at 18 °C for the production of the recombinant peptide in a soluble form. The bacterial cells were precipitated from the solution by centrifugation at 8000 rpm for 8 min. The hybrid protein that contained thioredoxin and HCRG21 peptide was isolated by the metal affinity chromatography on the Ni-NTA-agarose in the native condition from the soluble fraction of the cellular lysate after its ultrasound treatment according to the manufacturer’s instruction. Thioredoxin was cleaved by the treatment with cyanogen bromide according to the modified technique [73]. The recombinant HCRG21 peptide was purified by the reversed phase HPLC on a Jupiter C 4 column (10 × 250 mm, Phenomenex, Torrance, CA, USA), equilibrated by 0.1% TFA, pH 2.2, (Solution A), in gradient of acetonitrile (Solution B) concentration (0%–70%) for 70 min at 3 mL/min. The retention time of the target product was 31 min.

MALDI-TOF/MS spectrum of rHCRG21 was recorded using an Ultra Flex III MALDI-TOF/TOF mass spectrometer (Bruker, Bremen, Germany) with a nitrogen laser (SmartBeam, 355 nm), reflector and potential LIFT tandem modes of operation. Sinapinic acid was used as a matrix. An external calibration was employed using a polypeptide sample [17] with *m*/*z* 6107 and its doubly-charged variant at *m*/*z* 3053.

Homology sequence analysis was carried out using protein databases and BLAST programs [74].

The nucleotide sequence data of *hcrg21* reported in this paper will appear in the GenBank under the accession numbers KX900498. The accession numbers of the peptide sequences are InhVJ (UniProt KB P0DMJ5); Jn-IV (UniProt KB P16344); APHC1, APHC2, and APHC3 (UniProt KB B2G331, C0HJF4, and C0HJF3, respectively); ShPI-1 (UniProt P31713); AsKC1, AsKC2, and AsKC3 (UniProt KB Q9TWG0, Q9TWF9, and Q9TWF8, respectively); α-DTX, DTX-I, and DTX K (UniProt KB P00980, P00979, and P00982, respectively); and BPTI (Uniprot 00974).

### 3.4. Phylogenetic Analysis

Phylogenetic analysis of the known sea anemone Kunitz-type protease inhibitors was performed using the neighbor-joining (NJ) method with Poisson correction [75,76] in the MEGA 6 [56]. The bootstrap was performed with 1000 replicates. BPTI was used as an out-group.

### 3.5. Inhibitory Activity 

The trypsin/α-chymotrypsin inhibitory activity of the peptide was tested through the standard procedure [55] using *N*-α-benzoyl-d,l-arginine p-nitroanilide hydrochloride (BAPNA) and ethyl ester of *N*-benzoyl-l-tyrosine (BTEE) as a substrates, respectively. Determination of the trypsin/α-chymotrypsin inhibition constants of rHCRG21 was performed according to the method of Dixon [77] using substrates concentrations of 133 and 65 mM. The concentration of both enzymes in the reaction mixture was 215 nM. Concentrations of the tested peptide ranged from 0 up to 3.2 mM. The constants were calculated based on the results of three parallel experiments. Computational error limits are in the range of 0.1%–0.5%.

### 3.6. Expression of Voltage-Gated Ion Channels in Xenopus laevis Oocytes

For the expression of rKv1.1, hKv1.3, rKv1.4, rKv1.5, rKv1.6, Shaker IR, hERG, and TRPV1 in *Xenopus* oocytes, the linearized plasmids were transcribed using the T7 or SP6 mMESSAGE-mMACHINE transcription kit (Ambion, Austin, TX, USA). The harvesting of stage V–VI oocytes from anaesthetized female *Xenopus laevis* frog was previously described [78]. Oocytes were injected with 50 nL of cRNA at a concentration of 1 ng/nL using a micro-injector (Drummond Scientific, Broomall, PA, USA). The oocytes were incubated in a solution containing (in mM): NaCl, 96; KCl, 2; CaCl_2_, 1.8; MgCl_2_, 2 and HEPES, 5 (pH 7.4), supplemented with 50 mg/L gentamicin sulfate.

### 3.7. Electrophysiological Recordings

Two-electrode voltage-clamp recordings were performed at room temperature (18–22 °C) using a Geneclamp 500 amplifier (Molecular Devices, Sunnyvale, CA, USA) controlled by a pClamp data acquisition system (Axon Instruments, Foster City, CA, USA). Whole cell currents from oocytes were recorded 1–4 days after injection. Bath solution composition was ND96 (in mM): NaCl, 96; KCl, 2; CaCl_2_, 1.8; MgCl_2_, 2, and HEPES, 5 (pH 7.4). Voltage and current electrodes were filled with 3 M KCl. Resistances of both electrodes were kept between 0.8 and 1.5 MΩ. The elicited currents were filtered and sampled at, respectively, 1 kHz and 10 kHz (for potassium currents) and at, respectively, 0.2 kHz and 0.5 kHz (for TRPV1 currents), using a four-pole low-pass Bessel filter. Leak subtraction was performed using a –P/4 protocol. K_V_1.1–K_V_1.6 and *Shaker* currents were evoked by 500 ms depolarizations to 0 mV followed by a 500 ms pulse to −50 mV, from a holding potential of −90 mV. Current traces of hERG channels were elicited by applying a +40 mV prepulse for 2 s followed by a step to −120 mV for 2 s. TRPV1 currents were measured in ND96 solution using a protocol of −90 mV during 400 s. The recording chamber was perfused at a rate of 2 mL·min^−1^ with a ND96 solution. As previously described [79], capsaicin (2 μM) was used as an agonist and capsazepine (10 μM) as an antagonist of TRPV1. Capsaicin and capsazepine were purchased from Sigma. All data represent at least 3 independent experiments (*n* ≥ 3) in which independent experiments represent experiments on different cells expressing TRPV1 channel and are presented as mean ± standard error.

### 3.8. Homology Modeling and Docking

Model of HCRG21 peptide was built with methods of homology modeling. A serine protease inhibitor ShPI-1 of the *S. helianthus* sea anemone obtained from Protein Data Bank (PDB ID 1SHP) was used as a template. Structure model of peptide HCRG21 was generated with MOE 15.1001 CCG^®^ software [80]. Missing loops [61] in Frozen rTRPV1 structures, including a short fragment of 503–507 and the longer extracellular loop 3 of 604–626 of the pore domain have been reconstructed by Loop Modeler Feature in the program MOE 15.1001, for each loop 10 models as well as models of the structure of the extracellular and transmembrane domains of the receptor for all the simulated loop configurations were generated. The models quality was evaluated with the MOE 15.1001 software and PROCHECK [81] server. Since the receptor structures obtained by cryoelectron microscopy (3J5Q, 3J5R, 5IRZ, 5IRX, 5IS0) omitted more than 110 N-terminal and 71 C-terminal residues that form regulatory receptor domain and provide engagement TRPV1 interaction with inhibitory proteins (calmodulin) [82] and other intracellular partners, our TRPV1 model is adequate for transmembrane, as well as extracellular domains, including extracellular loops 2 and 3.

Modeling of macromolecular complexes was performed with the molecular docking technique for each TRPV1 structural model in a capsaicin-bound state with loops refined. Docking procedure was performed with ClusPro 2.0 server [83,84] and Protein-Protein Docking Feature of MOE 15.1001 software. All computed docking poses were clustered and analyzed. Among top-ranked docking solutions there were poses corresponding both to the peptide binding site localized at the transmembrane helix regions and at the cytoplasmic part of the channel, analogous to what has been reported for APHC1 [32]. However, these solutions were not selected for further analysis due to a lack of information about how Kunitz peptides insert into the lipid membrane. Concerning the intracellular part of TRPV1, the lack of new knowledge about regulatory domain structure permits us to employ receptor structural model [61] as reported earlier [32] for examine putative interaction mode of HCRG21 with this receptor portion. The most energetically favorable of highly populated and satisfied the constraints derived from in vitro or in vivo experiments (but non top-ranked) were underwent MD simulations and analyzed in more details. MD simulations of the HCRG21–TRPV1 complex in a hydrated POPC lipid bilayer performed in an Amber12:EHT force field [85] were performed under conditions of constant pressure, 300 K, and pH 7.0 using MOE 15.1001 software [80] for 100 ns. Prior to MD simulations, whole system was equilibrated to reduce initial bad contacts. Equilibration consisted in energy minimization of the initial side chains position with fixed backbone atoms, followed by a minimization with restrained carbon alpha atoms and a short molecular dynamic (100 ps). Computer simulation and theoretical studies were performed using cluster CCU “Far Eastern computing resource” FEB RAS (Vladivostok). Analysis of the contact surfaces of theoretical complexes and contribution of various amino acid residues to the formation of intermolecular interface was performed with MOE 15.1001 software [80] and Discovery studio 4.5 Visualizer [86] software. Visualization of the TRPV1–HCRG21 complex was performed with a Discovery Studio Visualizer BIOVIA^®^ software [86].

## 4. Conclusions

Kunitz-type peptides attract an increasing attention of investigators because of their important and intriguing role in many pathophysiological processes. Consequently, these pharmacologically interesting peptides are intensively being studied as potential drugs for treatment of inflammatory diseases and pain relief. Electrophysiological data show that the Kunitz-type peptide rHCRG21, belonging to the *H. crispa* multigene HCRG peptide subfamily, strongly inhibits the TRPV1 channel. The HCRG21–TRPV1 molecular docking and MD simulations allow to hypothesize the existence of two feasible molecular mechanisms of peptide–channel interactions: (i) pore-blocking as a result of extracellular application (independent of the presence or absence of capsaicin); and (ii) an intercellular binding with the regulatory channel domain (with three to four subunits) that triggers conformational changes of the pore helix region structure. Both these possible mechanisms resulted in an obstruction of the ion flow through TRPV1. At this moment, there is no direct evidence of Kunitz-type peptides permeating through the membrane and the hereupon following intercellular interaction with TRPV1. Clearly, more experiments are needed to verify the mechanism by which HCRG21 inhibits the TRPV1 channel. Identification of the key residues of HCRG21, together with site-directed mutagenesis of the channel, is required. In this work, we report the first venom derived peptide compound that is a full antagonist of the pain signal transmission through TRPV1. HCRG21 represents a promising tool for studying TRPV1 channels and, moreover, might be an interesting lead compound for the development of novel analgesics.

## Figures and Tables

**Table 1:** Physical and chemical characteristics of some native sea anemone BPTI/Kunitz-type canonical inhibitors and rHCRG21.

Sea Anemone	Peptide	Mr, Da	P1 Residue	*K_i_*, M	
				Trypsin	α-Chymotrypsin
*Bos taurus*	BPTI [55]		K	6.0 × 10^−14^	1.8 × 10^−13^
*A. sulcata*	AsKC1 [7]	6685 *	R	<3 × 10^−10^	n.d.
	AsKC2 [7]	6772 *		<3 × 10^−10^	n.d.
	AsKC3 [7]	6732 *		<3 × 10^−10^	n.d.
	SA5 II [13]	6937 **		3.0 × 10^−10^	n.d.
*A. elegantissima*	APEKTx1 [9]	7469 *		1.2 × 10^−7^ *	n.d.
*S. helianthus*	SHPI-1 [23]	6110 *	K	1.1 × 10^−10^	2.3 × 10^−9^
	SHPI-2 [23]	6195 **		3.8 × 10^−10^	n.d.
*H. crispa*	HCRG1 [18]	6196 *		2.8 × 10^−8^	n.d.
	HCRG2 [18]	6148.7 *		5.0 × 10^−8^	n.d.
*H. crispa*	rHCRG21	6228 *	T	2.0 × 10^−7^	7.0 × 10^−7^
	Jn-IV [14]	6165 *		9.6 × 10^−9^	n.d.
	InhVJ [17]	6106 *		7.38 × 10^−8^	9.93 × 10^−7^
	APHC1 [28]	6187 *		1.0 × 10^−6^	5.0 × 10^−6^
	APHC2 [29]	6185 *		9.0 × 10^−7^	4.5 × 10^−6^
	APHC3 [29]	6111 *		5.0 × 10^−7^	7.0 × 10^−6^

* MALDI-TOF/MS analysis data; ** UniProt data; n.d.: not determined.

## Supplementary Materials

The following are available online at www.mdpi.com/1660-3397/14/12/229/s1, Table S1: Oligonucleotide primers used and PCR cycling conditions for amplification and *hcrg21* gene synthesis, Table S2: Non-bonded interactions of HCRG21 in complex with intracellular (regulatory) domain of TRPV1 receptor, Table S3: Non-bonded interactions of HCRG21 in complex with extracellular domain of TRPV1 receptor.
