# Supplementary material for: Kunitz-Type Peptide HCRG21 from the Sea Anemone Heteractis crispa Is a Full Antagonist of the TRPV1 Receptor
Source: Mar Drugs. 2016 Dec 15;14(12):229. doi: 10.3390/md14120229 (PMC5192466; doi:10.3390/md14120229)
Supplement: Supplementary file 1 [file marinedrugs-14-00229-s001.doc]

Supplementary Materials: Kunitz-Type Peptide HCRG21 from the Sea Anemone *Heteractis crispa* Is a Full Antagonist of the TRPV1 Receptor

Margarita Monastyrnaya, Steve Peigneur, Elena Zelepuga, Oksana Sintsova, Irina Gladkikh, Elena Leychenko, Marina Isaeva, Jan Tytgat and Emma Kozlovskaya

**Table S1.** Oligonucleotide primers used and PCR cycling conditions for amplification and *hcrg21* gene synthesis.

| **Primer’s  Name** | **Oligonucleotide Sequence, 5’ → 3’** | **Round** | **PCR Cycling Conditions** |
| --- | --- | --- | --- |
| PCR amplification | | | |
| Inh_RG_F1 | atcaaaaccactcaagcccgtggca | 1 | 2 min 94 °C, 27 × (30 s 94 °C, 30 s  68 °C, 1 min 72 °C), 7 min 72 °C |
| Inh_Rev | actcgagttacgccctgcatatagctcggcat |
| Inh_RS_F2 | acgaattcatgcgtggcatttgttca | 2 | 2 min 94 °C, 27 × (30 s 94 °C, 30 s  68 °C, 30 s 72 °C), 7 min 72 °C |
| Inh_Rev | actcgagttacgccctgcatatagctcggcat |
| Synthesis of *hcrg21* gene | | | |
| RG21-D1 | gaaccgaaagttgttggtccgtgcaccgcttacttccgtcgtttct | 1 | 2 min 94 °C, 16 × (20 s 94 °C, 20 s  50 °C, 30 s 72 °C), 5 min 72 °C |
| RG21-D2 | acttcgactctgaaaccggtaaatgcaccccgttcatctacggtgg |
| RG21-D3 | ttgcgaaggtaacggtaacaacttcgaaaccctgcgtgcttgccgt |
| RG21-R1 | gtttcagagtcgaagtagaaacgacggaagta |
| RG21-R2 | ccgttaccttcgcaaccaccgtagatgaac |
| RG21-S1 | ggaattcc**atg**cgtggtatctgctctgaaccgaaagttgttgg ^*^ | 2 | 2 min 94 °C, 25 × (20 s 94 °C, 20 s  54 °C, 30 s 72 °C), 5 min 72 °C |
| RG21-Rev | tcctcgag**tca**agcacggcagatagcacggcaagcacgcag ^**^ |

* EcoRI restriction enzyme site are underlined, Met-codon for BrCN cleavage are bolded; ** XhoI restriction enzyme site are underlined, start and stop codons are bolded.

**Table S2.** Non-bonded interactions of HCRG21 in complex with intracellular (regulatory) domain of TRPV1 receptor.

| **Interaction Partners** | **Distance, Å** | **Interaction Type** |
| --- | --- | --- |
| B:LYS715:HZ2–HCRG21:GLU6:OE1 | 1.31118 | Hydrogen Bond; Electrostatic |
| B:LYS725:HZ1–HCRG21:GLU45:OE2 | 1.33864 | Hydrogen Bond; Electrostatic |
| HCRG21:ARG1:HT2–B:GLU812:OE2 | 1.35474 | Hydrogen Bond; Electrostatic |
| HCRG21:ARG51:HH11–D:ASP737:OD2 | 2.70113 | Hydrogen Bond; Electrostatic |
| HCRG21:ARG51:HH21–D:ASP734:OD2 | 2.67502 | Hydrogen Bond; Electrostatic |
| HCRG21:ARG51:HH22–D:ASP737:OD2 | 1.27995 | Hydrogen Bond; Electrostatic |
| HCRG21:ARG55:HH22–D:CYS742:SG | 2.19568 | Hydrogen Bond; Electrostatic |
| HCRG21:LYS8:NZ–D:CYS716:SG | 5.00493 | Electrostatic |
| HCRG21:ARG48:NH1–D:ASP734:OD1 | 5.39175 | Electrostatic |
| HCRG21:ARG51:NH1–D:ASP737:OD1 | 5.42851 | Electrostatic |
| A:ARG718:HH22–HCRG21:THR14:OG1 | 1.49773 | Hydrogen Bond |
| B:LYS715:HN–HCRG21:GLU38:O | 1.64787 | Hydrogen Bond |
| B:LYS715:HN–HCRG21:GLY39:O | 2.69985 | Hydrogen Bond |
| B:LYS715:HZ1–HCRG21:GLY39:O | 1.42102 | Hydrogen Bond |
| B:ARG718:HH12–HCRG21:GLU38:OE1 | 1.54930 | Hydrogen Bond |
| B:ARG718:HH22–HCRG21:GLU38:OE2 | 1.39913 | Hydrogen Bond |
| B:GLN728:HE22–HCRG21:GLU45:OE1 | 2.06398 | Hydrogen Bond |
| B:GLU762:HE2–HCRG21:SER5:O | 1.56520 | Hydrogen Bond |
| D:LYS719:HZ1–HCRG21:LYS28:NZ | 1.53579 | Hydrogen Bond |
| D:TRP741:HE1–HCRG21:THR26:O | 1.68905 | Hydrogen Bond |
| HCRG21:ARG1:HE–B:GLN817:OE1 | 2.12462 | Hydrogen Bond |
| HCRG21:ARG1:HH11–B:GLN817:OE1 | 1.65701 | Hydrogen Bond |
| HCRG21:ARG1:HH12–B:ASN756:O | 2.06388 | Hydrogen Bond |
| HCRG21:ARG1:HH22–B:ASN756:O | 1.76988 | Hydrogen Bond |
| HCRG21:GLY2:HN–B:GLU812:OE2 | 2.69190 | Hydrogen Bond |
| HCRG21:SER5:HG–B:GLU812:OE1 | 1.63384 | Hydrogen Bond |
| HCRG21:TYR16:HH–C:ARG718:O | 1.46590 | Hydrogen Bond |
| HCRG21:ARG18:HE–C:LYS719:O | 2.99472 | Hydrogen Bond |
| HCRG21:ARG18:HH21–C:LYS719:O | 1.50692 | Hydrogen Bond |
| HCRG21:CYS29:HN–D:ASP738:OD1 | 1.37528 | Hydrogen Bond |
| HCRG21:GLU38:HE2–B:LYS715:O | 1.35724 | Hydrogen Bond |
| HCRG21:ARG48:HH11–D:VAL729:O | 1.61064 | Hydrogen Bond |
| HCRG21:ARG48:HH22–D:VAL729:O | 3.00480 | Hydrogen Bond |
| HCRG21:ARG51:HE–D:ASP734:OD2 | 1.32288 | Hydrogen Bond |
| HCRG21:ARG51:HH21–D:PRO733:O | 2.02195 | Hydrogen Bond |
| HCRG21:ARG55:HE–D:CYS742:SG | 1.89049 | Hydrogen Bond |
| A:LYS711:HE1–HCRG21:PRO12:O | 2.97862 | Hydrogen Bond |
| A:LYS711:HE2–HCRG21:PRO12:O | 3.05711 | Hydrogen Bond |
| B:LEU714:HA–HCRG21:GLY39:O | 2.68745 | Hydrogen Bond |
| HCRG21:SER5:HB2–B:GLU812:OE1 | 3.00271 | Hydrogen Bond |
| HCRG21:LYS28:HA–D:ASP738:OD1 | 2.96962 | Hydrogen Bond |
| HCRG21:THR30:HB–D:ARG718:O | 2.78645 | Hydrogen Bond |
| HCRG21:PRO31:HD2–D:LYS719:O | 2.71659 | Hydrogen Bond |
| HCRG21:ARG19:NH2–B:PHE721 | 4.04008 | π-Cation; π-Donor Hydrogen Bond |
| HCRG21:ARG48:NH1–D:TYR731 | 4.50585 | Electrostatic |
| HCRG21:ARG19:HE–B:PHE721 | 2.95907 | π-Donor Hydrogen Bond |
| B:LEU727–HCRG21:ILE53 | 5.30992 | Hydrophobic, Alkyl |
| B:ILE760–HCRG21:ILE3 | 5.44712 | Hydrophobic, Alkyl |
| B:CYS767–HCRG21:PRO7 | 4.73873 | Hydrophobic, Alkyl |
| D:CYS716–HCRG21:LYS8 | 4.68698 | Hydrophobic, Alkyl |
| HCRG21:CYS4–B:VAL770 | 4.89148 | Hydrophobic, Alkyl |
| HCRG21:CYS13–A:LEU707 | 5.08160 | Hydrophobic, Alkyl |
| D:TRP741–HCRG21:LYS28 | 4.95420 | Hydrophobic, π-Alkyl |
| D:TRP741–HCRG21:LYS28 | 4.50311 | Hydrophobic, π-Alkyl |
| HCRG21:TYR16–C:ARG718 | 5.13846 | Hydrophobic, π-Alkyl |
| HCRG21:PHE22–B:VAL770 | 5.19733 | Hydrophobic, π-Alkyl |
| HCRG21:PHE22–D:CYS742 | 5.07516 | Hydrophobic, π-Alkyl |

**Table S3.** Non-bonded interactions of HCRG21 in complex with extracellular domain of TRPV1 receptor.

| **Interaction Partners** | **Distance, Å** | **Interaction Type** |
| --- | --- | --- |
| HCRG21:ARG1:HH12–C:GLU651:OE2 | 1.71568 | Hydrogen Bond; Electrostatic |
| HCRG21:ARG18:HH21–A:GLU651:OE1 | 3.09635 | Hydrogen Bond; Electrostatic |
| HCRG21:ARG48:HH11–B:GLU648:OE2 | 1.97853 | Hydrogen Bond; Electrostatic |
| HCRG21:ARG48:HH12–B:ASP646:OD2 | 2.31401 | Hydrogen Bond; Electrostatic |
| HCRG21:ARG48:HH21–B:GLU648:OE2 | 1.98169 | Hydrogen Bond; Electrostatic |
| HCRG21:ARG48:HH22–A:GLU636:OE2 | 1.77578 | Hydrogen Bond; Electrostatic |
| HCRG21:ARG51:HH22–D:GLU636:OE1 | 1.82154 | Hydrogen Bond; Electrostatic |
| C:LYS622:HZ2–HCRG21:ALA56:OXT | 1.87483 | Hydrogen Bond; Electrostatic |
| C:LYS639:HZ3–HCRG21:ALA56:O | 2.97200 | Hydrogen Bond; Electrostatic |
| HCRG21:ARG19:NH1–B:GLU651:OE2 | 5.39772 | Electrostatic |
| HCRG21:LYS28:NZ–D:ASP601:OD2 | 5.47147 | Electrostatic |
| HCRG21:LYS28:NZ–D:GLU651:OE2 | 4.41505 | Electrostatic |
| HCRG21:ARG48:NH1–A:GLU636:OE1 | 5.16541 | Electrostatic |
| HCRG21:ARG48:NH1–B:GLU648:OE1 | 4.97688 | Electrostatic |
| HCRG21:ARG48:NH2–B:ASP646:OD1 | 5.44845 | Electrostatic |
| HCRG21:ARG51:NH1–D:ASP646:OD1 | 4.32295 | Electrostatic |
| HCRG21:ARG55:NH1–C:ASP646:OD2 | 4.69303 | Electrostatic |
| HCRG21:ARG55:NH2–C:GLU636:OE1 | 4.77442 | Electrostatic |
| A:LYS622:NZ–HCRG21:GLU45:OE1 | 2.71769 | Electrostatic |
| B:LYS615:NZ–HCRG21:GLU38:OE2 | 2.73241 | Electrostatic |
| C:LYS615:NZ–HCRG21:GLU25:OE2 | 2.92558 | Electrostatic |
| C:ARG617:NH2–HCRG21:GLU25:OE2 | 3.66159 | Electrostatic |
| HCRG21:ARG1:H3–C:PHE649:O | 1.81189 | Hydrogen Bond |
| HCRG21:GLY2:H–C:CYS621:SG | 3.02215 | Hydrogen Bond |
| HCRG21:SER5:HG–C:GLY618:O | 2.74505 | Hydrogen Bond |
| HCRG21:LYS8:HZ2–D:SER619:OG | 1.84983 | Hydrogen Bond |
| HCRG21:LYS8:HZ3–D:ARG617:O | 2.47015 | Hydrogen Bond |
| HCRG21:ARG19:HH21–A:ALA620:O | 2.82843 | Hydrogen Bond |
| HCRG21:ARG19:HH22–A:ALA620:O | 3.07112 | Hydrogen Bond |
| HCRG21:LYS28:HZ1–D:PHE649:O | 1.97819 | Hydrogen Bond |
| HCRG21:ARG48:HE–A:GLU636:OE1 | 2.12791 | Hydrogen Bond |
| HCRG21:ARG55:HH11–C:MET644:O | 2.59975 | Hydrogen Bond |
| HCRG21:ALA56:H–D:ASP646:OD1 | 1.80232 | Hydrogen Bond |
| A:CYS616:HG–HCRG21:TYR16:O | 1.98778 | Hydrogen Bond |
| C:ARG617:HH11–HCRG21:GLU25:O | 2.34598 | Hydrogen Bond |
| C:ARG617:HH12–HCRG21:GLU25:O | 2.51443 | Hydrogen Bond |
| D:SER619:HG–HCRG21:PRO31:O | 1.83696 | Hydrogen Bond |
| HCRG21:ARG1:HD2–C:GLU651:OE2 | 2.60149 | Hydrogen Bond |
| HCRG21:SER5:HB2–C:GLY618:O | 2.98581 | Hydrogen Bond |
| HCRG21:SER5:HB3–C:GLY618:O | 3.08901 | Hydrogen Bond |
| HCRG21:THR30:HB–D:ALA620:O | 2.53780 | Hydrogen Bond |
| HCRG21:PRO31:HD3–D:LYS622:O | 2.89746 | Hydrogen Bond |
| HCRG21:PHE32:HA–D:GLY618:O | 2.43269 | Hydrogen Bond |
| HCRG21:GLY36:HA2–A:SER619:OG | 2.63060 | Hydrogen Bond |
| HCRG21:ARG48:HD2–B:ASP646:OD2 | 2.64364 | Hydrogen Bond |
| HCRG21:ARG48:HD3–A:GLU636:OE1 | 3.00315 | Hydrogen Bond |
| HCRG21:ARG55:HA–D:ASP646:OD1 | 3.05952 | Hydrogen Bond |
| A:GLY618:HA2–HCRG21:GLY36:O | 2.78012 | Hydrogen Bond |
| C:ARG617:HD2–HCRG21:GLU25:OE2 | 2.84227 | Hydrogen Bond |
| D:SER619:HA–HCRG21:PRO31:O | 2.79356 | Hydrogen Bond |
| D:SER619:HB2–HCRG21:PRO31:O | 2.67477 | Hydrogen Bond |
| D:PRO623:HD2–HCRG21:CYS29:O | 2.45301 | Hydrogen Bond |
| HCRG21:LYS28:NZ–D:PHE649 | 4.67469 | Electrostatic, π-Cation |
| HCRG21:ARG51:NH2–D:PHE649 | 3.48042 | Electrostatic, π-Cation |
| HCRG21:ARG55:NH2–C:PHE649 | 3.70816 | Electrostatic, π-Cation |
| A:SER619:HB3–HCRG21:PHE17 | 2.37992 | Hydrophobic,π-Sigma |
| HCRG21:ALA15–A:CYS616 | 4.07655 | Hydrophobic, Alkyl |
| HCRG21:ARG51–D:MET644 | 4.63590 | Hydrophobic, Alkyl |
| HCRG21:ARG55–C:MET644 | 5.18104 | Hydrophobic, Alkyl |
| HCRG21:PHE17–A:CYS616 | 4.52386 | Hydrophobic, π-Alkyl |
| HCRG21:PHE17–A:CYS621 | 4.96167 | Hydrophobic, π-Alkyl |
| D:PHE649–HCRG21:LYS28 | 4.37228 | Hydrophobic, π-Alkyl |
